# Development of a novel ddPCR assay for the simultaneous detection of the protozoan parasites *Leishmania infantum* and *Leishmania tarentolae*

**DOI:** 10.1186/s13071-025-06871-3

**Published:** 2025-07-01

**Authors:** Alessandro Alvaro, Giulia Maria Cattaneo, Ilaria Varotto-Boccazzi, Riccardo Molteni, Jairo Alfonso Mendoza-Roldan, Matteo Brilli, Matilde Silvia Conconi, Virginia Giovagnoli, Alessandro Manenti, Domenico Otranto, Claudio Bandi, Sara Epis

**Affiliations:** 1 Department of Biosciences, University of Milan, 20133 Milan, Italy; 2 Department of Veterinary Medicine, University of Bari, Valenzano, 70010 Bari, Italy; 3 VisMederi Srl, 53100 Siena, Italy; 4 National PhD Programme in One Health Approaches to Infectious Diseases and Life Science Research, Department of Public Health, Experimental and Forensic Medicine, University of Pavia, 27100 Pavia, Italy; 5 Department of Veterinary Clinical Sciences, City University of Hong Kong SAR, Hong Kong, China

**Keywords:** Droplet digital PCR, *Leishmania infantum*, *Leishmania tarentolae*, Leishmaniasis, Epidemiology, Sand flies

## Abstract

**Background:**

Leishmaniases, caused by protozoan parasites of the genus *Leishmania*, are vector-borne diseases occurring mainly in the tropics and subtropics of the world, as well as in the Mediterranean Basin. In this area, the mammalian pathogen *Leishmania infantum* is endemic, along with the reptile-associated *Leishmania tarentolae*. The two species occur in sympatry, and there is evidence that the exposure to *L. tarentolae* in mammalian hosts may elicit a protective immune response towards pathogenic *Leishmania* species. Accurate detection methods for both species are therefore crucial for gathering comprehensive information on the epidemiology of leishmaniases. In microbiological diagnosis, limits in detection performance imply the risk of false negatives and other issues, which highlights the need for sensitive methods.

**Methods:**

Here, we developed a droplet digital polymerase chain reaction assay targeting the kinetoplast minicircle DNA, for the simultaneous and differential detection of *L. infantum* and *L. tarentolae*. The assay features primers designed to bind to both species and species-specific probes. The assay was validated on three cultured isolates for each species, whose cells were spiked into *Leishmania*-negative dog blood, and on *Leishmania*-positive sand flies. Sensitivity was assessed with testing serial dilutions, and specificity was evaluated by assessing the cross-reactivity of the probes with the controls of *Leishmania*-free dog blood and male sand fly DNA.

**Results:**

The assay demonstrated high sensitivity, with a limit of detection corresponding to one *Leishmania* cell in the reaction mix for isolates of both *L. infantum* and *L. tarentolae*. Limited cross-reaction of the *L. tarentolae*-targeting probe was observed on *L. infantum* isolates. No cross-reaction was observed with the controls of *Leishmania*-free dog blood and male sand flies.

**Conclusions:**

The protocol can represent a valuable method for comprehensive surveillance in both canine hosts and sand flies in areas in which *L. infantum* and *L. tarentolae* occur in sympatry.

**Graphical Abstract:**

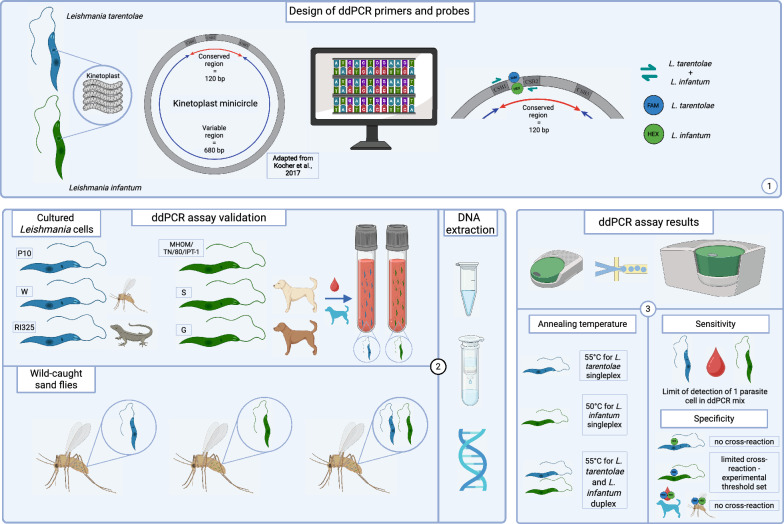

## Background

Leishmaniases are vector-borne diseases caused by protozoan parasites of the genus *Leishmania* (Kinetoplastida: Trypanosomatidae), which are transmitted to their vertebrate hosts via the bite of infected female sand flies (Diptera: Psychodidae: Phlebotominae) [1]. These diseases represent a significant public health burden in various parts of the world, particularly in tropical and subtropical regions, and are also endemic in the Mediterranean Basin [2].

In Italy, human cutaneous leishmaniasis (CL) and visceral leishmaniasis (VL), as well as canine leishmaniasis (CanL), are endemic and are caused by *Leishmania infantum* Nicolle, 1908 [3]. In the country, the main vectors of *L. infantum* are sand flies of the species *Phlebotomus perniciosus* Newstead, 1911 and *Phlebotomus perfiliewi* Parrot, 1930 [4, 5].

In addition, the reptile-associated *Leishmania tarentolae* Wenyon, 1921 has also been detected in Italy [5–9]. This parasite is regarded as non-pathogenic to humans and mammals in general, and is therefore exploited as an antigen production and vaccine development platform [10, 11]. The ecology and epidemiology of *L. tarentolae*, however, remain poorly understood. *Leishmania tarentolae* is vectored by sand flies of the species *Sergentomyia minuta* (Rondani, 1843), which feed mainly on reptiles [12]. Nevertheless, this parasite may circulate in vectors other than *S. minuta,* as *L. tarentolae* DNA has been detected in the sand fly *P. perniciosus* in Italy [6, 13]. This finding is in accordance with the development of *L. tarentolae* in *P. perniciosus* under laboratory conditions [14]. The parasite may also circulate in non-reptilian hosts, and evidence of this was gathered in central and southern Italy by molecular detection of *L. tarentolae* in dogs and humans [9, 13]. Moreover, *S. minuta* sand flies have been reported to feed on humans both in captivity [15] and in the wild [16, 17], and may therefore transmit *L. tarentolae* to non-reptilian hosts. This is particularly interesting given that exposure of mammalian hosts to *L. tarentolae* may elicit a protective immune response towards pathogenic *Leishmania* species [18]. On the other hand, in Italy, the DNA of *L. infantum* has been detected in sand flies of the species *S. minuta* [13, 19]. Furthermore, the DNA of both species of *Leishmania* has been detected simultaneously in sand fly vectors and vertebrate hosts in the country [8, 9]. In addition, in Italy, *S. minuta* and *P. perniciosus* have repeatedly been collected in the same sites, raising the potential occurrence of *L. infantum* and *L. tarentolae* in (i) sympatric conditions, (ii) unconventional vectors (e.g. *L. infantum* in *S. minuta* and *L. tarentolae* in *Phlebotomus* spp.), and iii) unconventional hosts (e.g. *L. infantum* in reptiles and *L. tarentolae* in mammals) [6, 9].

Therefore, accurately detecting both *L. infantum* and *L. tarentolae* in sand flies and vertebrate hosts is essential for reconstructing leishmaniasis epidemiological scenarios and for providing insights into the transmission dynamics of the infection. Traditional diagnostic methods, such as microscopy and polymerase chain reaction (PCR), may have limitations in terms of sensitivity, specificity, and quantification accuracy, particularly when dealing with complex biological samples containing low concentrations of parasite DNA [20]. For this reason, droplet digital PCR (ddPCR) has emerged as a powerful tool for the detection and quantification of nucleic acids with unparalleled sensitivity, specificity, and accuracy. By partitioning PCR reactions into thousands of individual droplets, ddPCR enables absolute quantification of target DNA molecules, overcoming many of the limitations associated with traditional PCR methods [20]. For this reason, ddPCR has been employed in the detection of a variety of pathogenic microorganisms [21–25]. Only three ddPCR protocols for the detection of *Leishmania* DNA have been published to date: the first protocol was developed for the detection of *Leishmania braziliensis* Vianna, 1911 DNA in samples obtained from patients affected by cutaneous leishmaniasis [26], the second for the quantification of *L. infantum* in dogs [27], and the third protocol was validated on six *Leishmania* isolates representing five species [28]. In addition, to the best of our knowledge, no ddPCR protocols have been developed and published for the screening of *Leishmania* spp. in sand fly vectors.

We developed a novel ddPCR assay targeting the minicircles of the kinetoplast DNA (kDNA) for the sensitive and specific simultaneous detection of *L. tarentolae* and *L. infantum* and validated this method on DNA samples derived from laboratory-maintained *Leishmania* strains, spiked to *Leishmania*-free dog blood, and from wild-caught sand flies. The method showed high sensitivity, allowing for the detection of low parasite loads, and high specificity, showing only minimal cross-reactivity.

## Methods

### Oligonucleotide design

A region encompassing the Conserved Sequence Blocks 1 and 2 (CSB 1 and CSB 2) of the kinetoplast minicircles was chosen as the ddPCR target. Sequences from *L. infantum*, *L. tarentolae*, *L. donovani* (Laveran et Mesnil, 1903), *L. major* Yakimoff et Schokhor, 1914, *L. tropica* Wright, 1903, and *L. braziliensis* Vianna, 1911 were aligned to design the primers and the probes. The primers were designed to amplify both *L. tarentolae* and *L. infantum* DNA, whereas the probes were designed to be species-specific. The resulting amplicon was 56 bp long. The absence of cross-reaction and formation of secondary structure of primers and probes was verified using the PrimerPooler software [29]. The probes were labelled with the FAM fluorophore for *L. tarentolae* and with the HEX fluorophore for *L. infantum*. All the primers and probes were manufactured by Eurofins Genomics (Konstanz, Germany). The oligonucleotide sequences are reported in Table 1.

**Table 1:** Oligonucleotides designed and employed for the validation of the ddPCR assay

Oligonucleotide name	Type	Target gene	Species	Sequence (5′-3′)
MC_Leish_F	Forward primer	Minicircles CSB 1 and CSB 2	*L. tarentolae* + *L. infantum*	TCCCAAACTTTTTAGGTC
MC_Leish_R	Reverse primer	Minicircles CSB 1 and CSB 2	*L. tarentolae* + *L. infantum*	GCRWTTTTGGCCAWTTTTTG
MC_tar_FAM	Probe FAM	Minicircles CSB 1 and CSB 2	*L. tarentolae*	CCGAAAACCGAAAAATGCATGCA
MC_inf_HEX	Probe HEX	Minicircles CSB 1 and CSB 2	*L. infantum*	GCGAAAACSGAAAAATGGGTGCA

### ddPCR experiment conditions

Each ddPCR reaction targeting *L. tarentolae* and *L. infantum* minicircles included 11 μL of Bio-Rad ddPCR Supermix for Probes (No dUTP) (Bio-Rad, Hercules, CA, USA) primers at a final concentration of 900 nM and probes at a final concentration of 250 nM, 0.78 μL of Milli-Q water, and 5 μL of DNA. The assays were performed in the QX200 Droplet Digital PCR System (Bio-Rad, Hercules, CA, USA). The PCR reaction was conducted on a SimpliAmp Thermal Cycler (Applied Biosystems, Waltham, MA, USA) following a thermal protocol of 10 min at 95 °C for denaturation, 50 cycles of annealing at 50 °C, and elongation at 72 °C. Annealing temperatures were chosen after gradient PCR experiments (see below).

### Dog blood handling

The methods to handle dog blood, which is derived from a previous study [30], were approved by the Clinvet Institutional Animal Care and Use Committee (no. CG1331-CVMO22/216). All experiments involving the use of dog blood were performed in accordance with the ARRIVE guidelines and international regulations. All methods were carried out in accordance with relevant guidelines and regulations.

### ddPCR assay validation

#### *Leishmania*-cultured cells spiked in dog blood

The ddPCR assay validation was performed on DNA extracted from both *L. tarentolae* and *L. infantum* cultures spiked into *Leishmania*-negative dog blood, derived from another study [30]. For *L. tarentolae*, the commercial P10 strain (Jena Bioscience, Jena, Germany), maintained in Brain Heart Infusion (BHI) broth, was used. Alongside the P10 strain, the DNA of wild *L. tarentolae* strains “W”, isolated from the sand fly vector *S. minuta*, and “RI325”, isolated from the reptile host *Tarentola mauritanica* (Linnaeus, 1758), were used. Both wild strains were isolated at the Parasitology and Micology laboratory of the University of Bari Aldo Moro and were maintained in Schneider broth. For *L. infantum*, the DNA of the MHOM/TN/80/IPT-1 reference strain (Office International des Epizooties [OIE] Reference Laboratory National Reference Center for Leishmaniasis—C.Re.Na.L., Palermo, Italy) and the “S” and “G” strains recently isolated from dogs from southern Italy were used. The dog strains were isolated at the University of Bari Aldo Moro and maintained in Schneider’s *Drosophila* medium.

*Leishmania* cells were counted in a counting chamber under a Leica DM300 optical microscope (Leica Microsystems, Germany) and then spiked to 100 µl of *Leishmania*-negative dog blood. Amounts of 10^6^, 5 × 10^5^, 10^5^, 5 × 10^4^, 10^4^, 5 × 10^3^, 10^3^, 5 × 10^2^, 10^2^, 50, and 20 cells were used. These quantities correspond to 10^4^, 5 × 10^3^, 10^3^, 5 × 10^2^, 10^2^, 50, 10, 5, 1, 0.5, and 0.2 cells/µl of blood, respectively. The mixture was then subjected to DNA extraction using the DNeasy Blood and Tissue Kit (Qiagen, Hilden, Germany). The elution step was carried out in 100 μl, and 5 μl of DNA was added to the ddPCR mix. This means that the estimated *Leishmania* DNA present in the mix should correspond to 1/20 of the DNA of the original cell quantities, namely to 5 × 10^4^, 25 × 10^3^, 5 × 10^3^, 25 × 10^2^, 500, 250, 50, 25, 5, 2.5, and 1 cell, respectively.

The annealing temperatures of the assay thermal protocol were selected based on gradient PCR experiments, with temperatures ranging from 45 °C to 60 °C. Each of the isolated serial dilutions was run in triplicate.

### Cross-reactivity testing

The potential of cross-reactivity of the probes was assessed by testing the DNA extracted from the dog blood which *Leishmania* cells where spiked to, and the DNA extracted from four male sand flies, collected during routine monitoring activity carried out by laboratory members.

### Wild-caught sand flies

Fifteen female sand fly DNA samples from the collection of the Parasitology and Micology laboratory of the University of Bari Aldo Moro were used to validate the ddPCR assay (n. 13 = *S. minuta*; n. 2 = *P. perniciosus*). These sand flies have been collected in the Valenzano municipality, Bari district. Their DNA samples have been previously screened for both *L. tarentolae* and *L. infantum* with a duplex quantitative real-time PCR (qPCR) assay targeting the first Internal Transcribed Spacer (ITS-1) region [31].

### Statistical analyses

All the statistical analyses of the study were carried out in the R programming environment (https://www.r-project.org/).

## Results

### ddPCR annealing set-up

**Fig. 1:**
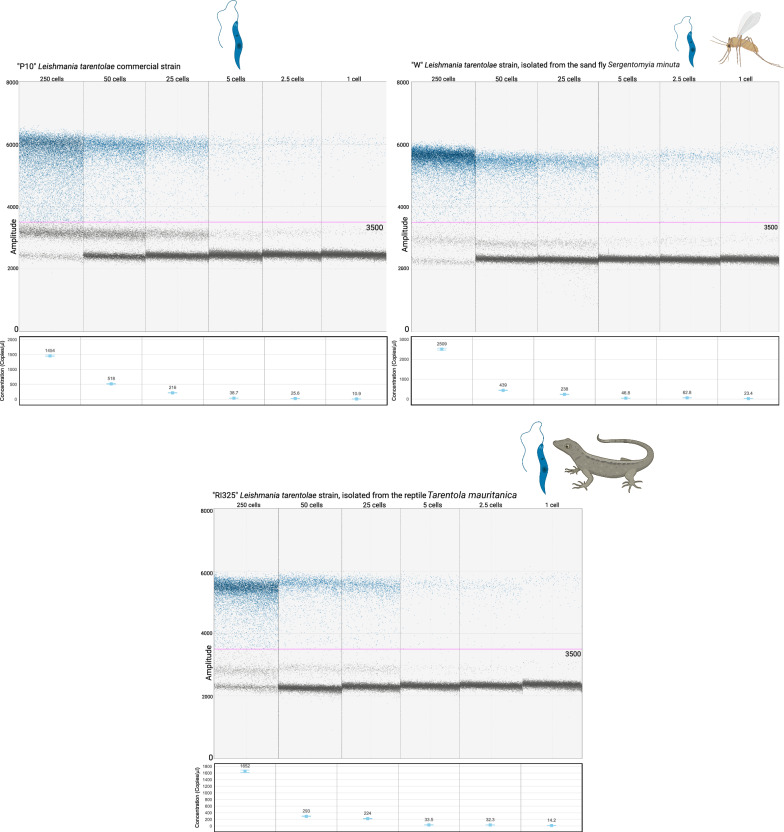
ddPCR assay sensitivity assessment on dog blood spiked with serial dilutions of the commercial P10 *L. tarentolae* strain (Jena Bioscience), and the wild “W” and “RI325” *L. tarentolae* strains. Annealing temperature of 55 °C. The limit of detection is 0.2 *Leishmania* cells/μl of DNA extracted from dog blood added to the ddPCR mix, corresponding to one *Leishmania* cell present in the ddPCR mix. The fluorescence amplitude intensity is indicated on the *y*-axis. Each dot represents an individual droplet. The pink line represents the amplitude threshold above which a droplet is assigned as positive (i.e. it contains one or more target copies, coloured in blue), and below which a droplet is assigned as negative (i.e. it contains no target copies, coloured in grey). For each sample, the number of *Leishmania* cells per μL of input DNA sample is shown above, while the concentration of target DNA copies per μL of input DNA sample is displayed in the box below

The optimal annealing temperature for detecting *L. tarentolae* and *L. infantum* was determined through experimentally established gradients using *Leishmania*-free dog blood spiked with *Leishmania* cells coming from the six above-mentioned laboratory-maintained strains (see Methods section). For *L. tarentolae*, the best amplification performance was obtained at 55 °C. A non-specific population of droplets was present at 3500 Relative Fluorescence Units (RFUs) amplitude but was clearly distinguishable from the droplets of interest, which form at around 5500–6000 RFUs amplitude (Fig. 1). “Rain” was observed between the non-specific population and the droplets of interest.

**Fig. 2:**
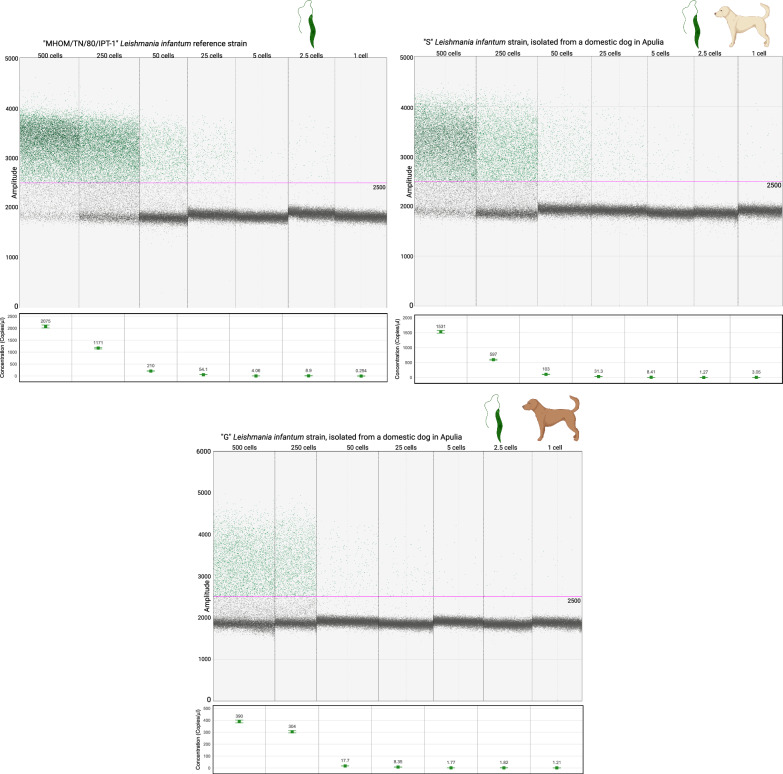
ddPCR assay sensitivity assessment on dog blood spiked with serial dilutions of the *L. infantum* “MHOM/TN/80/IPT-1” reference strain (OIE Reference Laboratory National Reference Center for Leishmaniasis—C.Re.Na.L., Palermo, Italy), and the wild “S” and “G” *L. infantum* strains (University of Bari Aldo Moro). Annealing temperature of 50 °C. The limit of detection is 0.2 *Leishmania* cells/μl of DNA extracted from dog blood added to the ddPCR mix, corresponding to one *Leishmania* cell present in the ddPCR mix. The fluorescence amplitude intensity is indicated on the *y*-axis. Each dot represents an individual droplet. The pink line represents the amplitude threshold above which a droplet is assigned as positive (i.e. it contains one or more target copies, coloured in green), and below which a droplet is assigned as negative (i.e. it contains no target copies, coloured in grey). For each sample, the number of *Leishmania* cells per μL of input DNA sample is shown above, while the concentration of target DNA copies per μL of input DNA sample is displayed in the box below

As for *L. infantum* detection, the best amplification performance was obtained at 50 °C instead of 55 °C. The maximum density of droplets in positive samples was localized between 3000 and 4000 RFUs, with more “rain” observed compared to the *L. tarentolae* isolates. However, the positive droplets were distinguishable from the negative droplets occurring at around 2000 RFUs. A statistically significant difference was observed after comparing the concentrations of target DNA obtained from the above-mentioned serial dilutions of *L. infantum* MHOM/TN/80/IPT-1 reference strain at 50 °C and 55 °C of annealing (Shapiro–Wilk test for normality *P*-value = 0.004754; Wilcoxon signed rank exact test, *P*-value = 0.01563). In summary, the annealing temperature of 55 °C resulted to be too high for *L. infantum*, and a temperature of 50 °C was chosen instead (Fig. 2). When testing *L. tarentolae* samples at 50 °C annealing, no significant differences were observed with regard to the results obtained at 55 °C, considering the above-mentioned serial dilutions of the *L. tarentolae* P10 isolate (Shapiro–Wilk test for normality *P*-value = 0.006; Wilcoxon signed rank exact test, *P*-value = 0.1563). The only difference was observed in the droplets’ amplitude: at 50 °C annealing, positive droplets showed an amplitude of ~ 8000 RFUs, while the non-specific population had an amplitude of 5000 RFUs. The positive and negative droplets remained clearly separated. Rain was still observed. In conclusion, for duplex application targeting both *Leishmania* species, we decided to set the annealing temperature at 50 °C.

### Assay sensitivity and specificity

The presented ddPCR assay showed high sensitivity. The assay limit of detection for both species was 0.2 *Leishmania* cells/μl of dog blood. In other words, the assay is able to detect the DNA of one *Leishmania* cell present in the ddPCR mix (0.2 cells/5 μl of DNA in the mix).

**Fig. 3:**
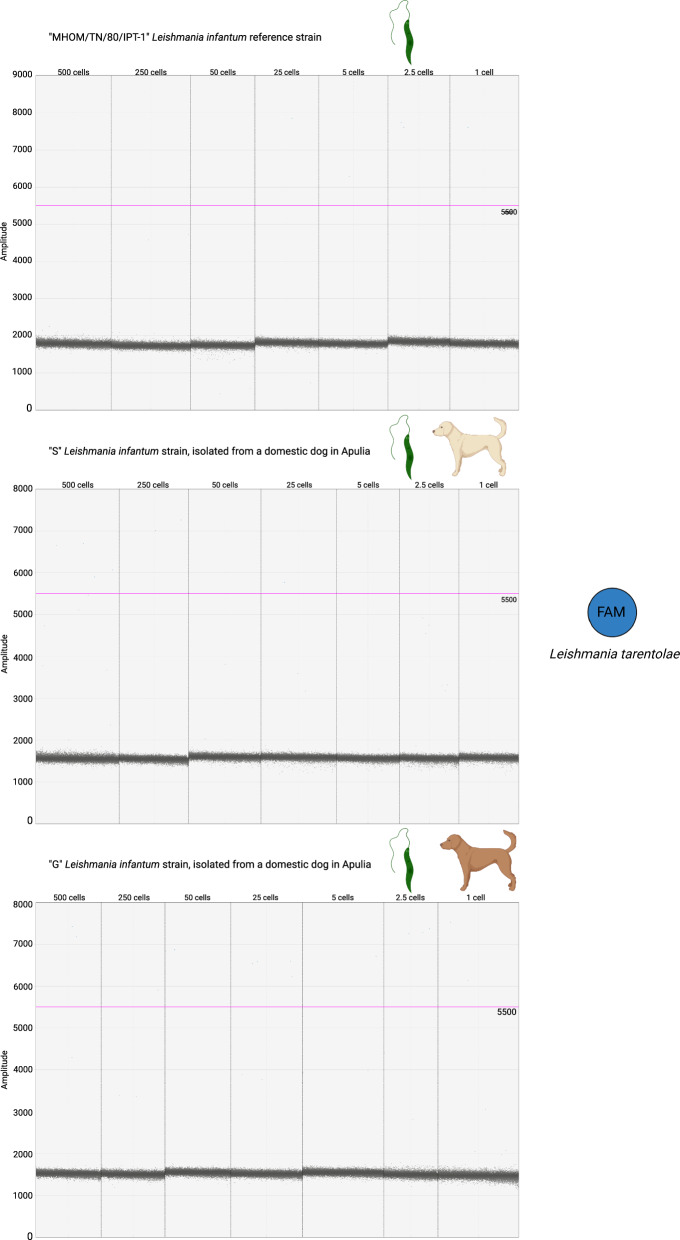
ddPCR specificity assessment by testing for cross-reaction between the FAM probe and the *L. infantum* DNA extracted from the three isolates used in the study. Annealing temperature of 50 °C. A maximum of four FAM-positive droplets were detected when analysing *L. infantum* samples. Therefore, any result with fewer than five FAM-positive droplets should be considered negative. The fluorescence amplitude intensity is indicated on the *y*-axis. Each dot represents an individual droplet. The pink line represents the amplitude threshold above which a droplet is assigned as positive (i.e. it contains one or more target copies, coloured in blue), and below which a droplet is assigned as negative (i.e. it contains no target copies, coloured in grey)

During the assay validation process, specificity was assessed by testing the *Leishmania*-spiked dog blood, inverting the probes (i.e. FAM included in the mix for *L. infantum* and HEX for *L. tarentolae*). No cross-reaction occurred for the HEX probe with regards to *L. tarentolae*-spiked blood, while one to four FAM-positive droplets were observed in *L. infantum*-spiked blood, regardless of the parasite concentrations (Fig. 3).

**Fig. 4:**
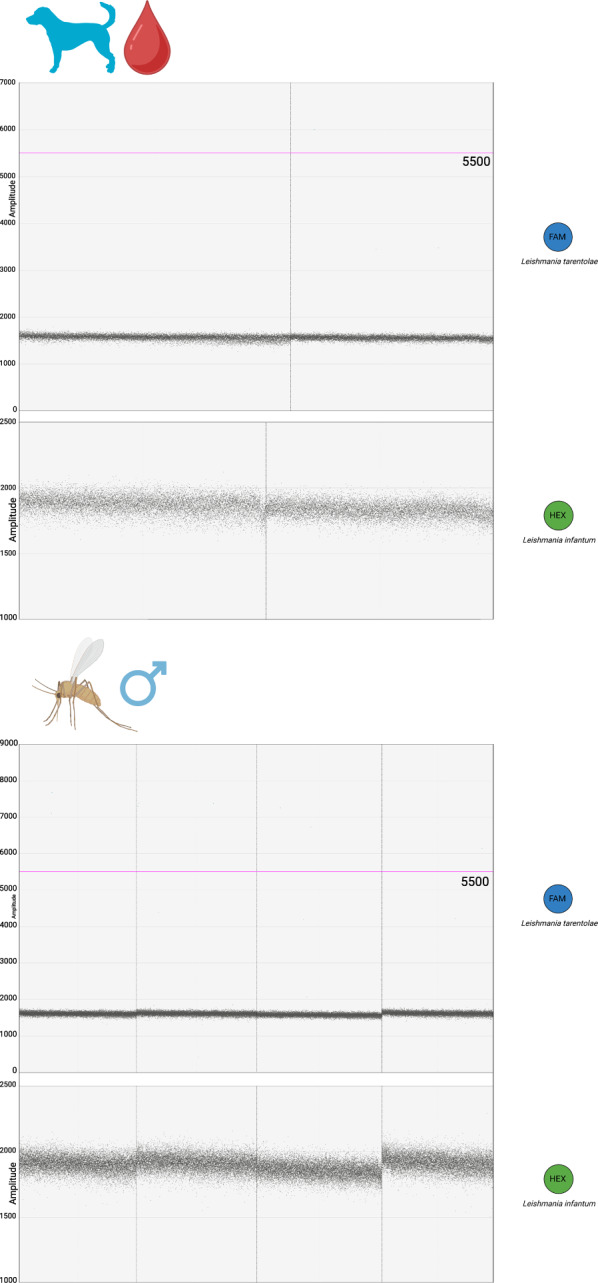
ddPCR assay specificity assessment on the controls represented by DNA extracted from *Leishmania*-negative dog blood and male sand flies. Annealing temperature of 50 °C. Each sample has been tested with both the FAM probe, targeting *L. tarentolae*, and with the HEX probe, targeting *L. infantum*. A maximum of two FAM-positive droplets have been observed in the sand fly samples, whereas no droplets hybridized with the HEX probe. Given the threshold set after testing for cross-reaction between the FAM probe and the *L. infantum* DNA, the control samples should be considered negative. For each sample, the FAM result is shown in the box on the top, while the HEX result is displayed in the box below. The fluorescence amplitude intensity is indicated on the *y*-axis. Each dot represents an individual droplet. The pink line represents the amplitude threshold above which a droplet is assigned as positive (i.e. it contains one or more target copies, coloured in blue), and below which a droplet is assigned as negative (i.e. it contains no target copies, coloured in grey)

The ddPCR assay also showed high specificity: the HEX probe, targeting *L. infantum*, showed no cross-reactivity with *Leishmania*-free dog blood. Regarding the FAM probe, targeting *L. tarentolae*, only a single droplet resulted positive at 50 °C of annealing while testing dog DNA (Fig. 4). While testing *Leishmania*-free male sand flies (see Methods section), the HEX probe targeting *L. infantum* did not hybridize with the DNA extracted from the insects. Considering the FAM probe, two positive droplets were detected in three out of four male DNA samples, while in the fourth one positive droplet was detected (Fig. 4). However, while testing DNA of female *L. infantum* positive sand flies (see next section) no cross reaction occurred between the FAM probe and the DNA of those samples.

The concentration, expressed as copies of target/µL, of the serial dilutions of the *L. tarentolae* “W” isolates and all *L. infantum* isolates did not exhibit a constant decrease between the samples of 1, 0.5, and 0.2 parasite cells/µL of dog blood. This may be due to the effect of chance or to dilution errors that can occur at such low cell concentrations.

### ddPCR vs ITS-1 qPCR

**Fig. 5:**
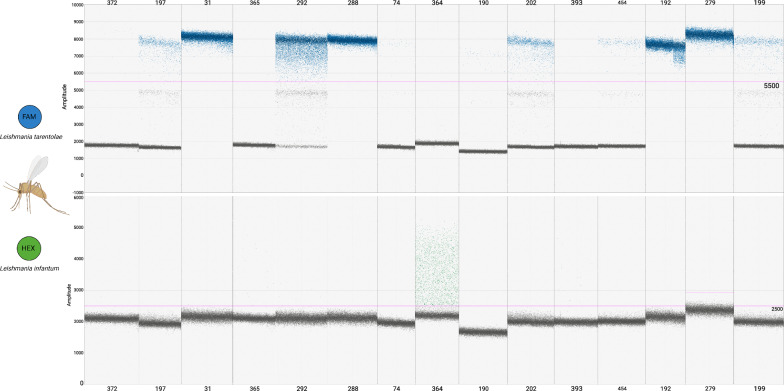
Results of the ddPCR testing of the qPCR-*Leishmania*-positive sand flies from the collection of the University of Bari Aldo Moro. Annealing temperature of 50 °C. Each sample has been tested with both the FAM probe, targeting *L. tarentolae*, and with the HEX probe, targeting *L. infantum*. For each sample, the FAM result is shown in the box on the top, while the HEX result is displayed in the box below. Sample names are indicated at the bottom of the HEX box. The fluorescence amplitude intensity is indicated on the *y*-axis. Each dot represents an individual droplet. The pink line represents the amplitude threshold above which a droplet is assigned as positive (i.e. it contains one or more target copies, coloured in blue or green), and below which a droplet is assigned as negative (i.e. it contains no target copies, coloured in grey)

The ddPCR, targeted to the minicircle, and the qPCR, targeted to the ITS-1 (see Methods section), showed congruence in their results on 11 out of 15 sand fly DNA samples (73.3%). Two *S. minuta* sand flies resulted positive to *L. tarentolae* only at qPCR testing, but positive to both *L. tarentolae* and *L. infantum* after the application of the ddPCR herein described. In addition, one *S. minuta* sand fly and one *P. perniciosus* sand fly showed positivity to *L. tarentolae* after qPCR, but ddPCR resulted positive to *L. infantum,* and negative to *L. tarentolae*. The ddPCR target amplicon concentrations and qPCR quantification cycles (cqs) are reported in Table 2. The ddPCR results are reported in Fig. 5.

**Table 2:**
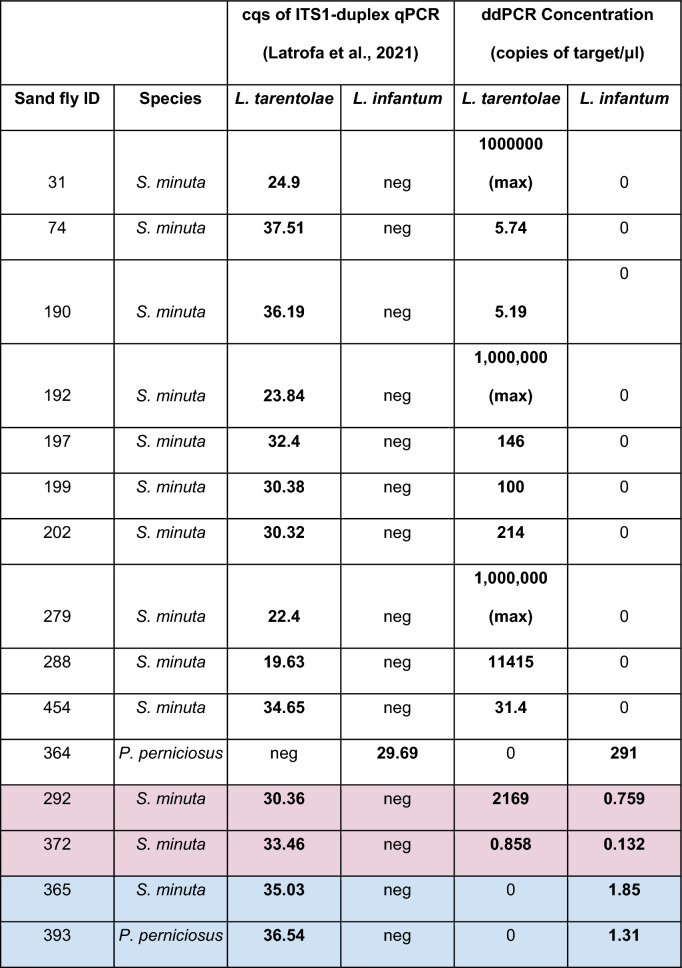
Results of ITS-1-targeted qPCR and ddPCR on the same female *Leishmania*-positive samples.

Samples whose corresponding cells are highlighted in red showed positivity to both species after ddPCR; samples whose corresponding cells are highlighted in blue were positive only for *L. infantum* after ddPCR, despite having shown positivity for *L. tarentolae* in the qPCR results

### Estimation of parasite number in sand flies

Comparing the concentrations obtained from ddPCR quantification of serial *Leishmania* cell dilutions in dog blood can provide a broad estimate of the number of parasite cells infecting the tested sand flies. Three individual sand fly females showed the maximum ddPCR concentration of 1,000,000 copies of amplified target DNA/μl. All the other samples ranged from less than one copy of target/μl to ~ 11,500 copies of target/μl. Based on these results, we estimated parasite numbers, which ranged from less than one to 5000 or more, in positive sand flies, as reported in Table 3. We emphasize that, in saturated samples (i.e. those with maximum ddPCR concentration of 100,000 copies), the number of parasite cells at 5000 could be an underestimation.

**Table 3:** Estimated number of parasite cells in relation to concentration values of “W” isolate for *L. tarentolae* and “G” isolate for *L. infantum*

Sand fly ID	Species	Estimated *L. tarentolae* cells	Estimated *L. infantum* cells
31	*S. minuta*	5000 and up	0
74	*S. minuta*	less than 1	0
190	*S. minuta*	less than 1	0
192	*S. minuta*	5000 and up	0
197	*S. minuta*	2.5—5 parasites	0
199	*S. minuta*	2.5—5 parasites	0
202	*S. minuta*	~ 25 parasites	0
279	*S. minuta*	5000 and up	0
288	*S. minuta*	Over 500	0
454	*S. minuta*	~ 1 parasite	0
364	*P. perniciosus*	0	50–250 parasites
292	*S. minuta*	~ 250 parasites	less than 1 parasite
372	*S. minuta*	less than 1 parasite	0
365	*S. minuta*	0	~ 1 parasite
393	*P. perniciosus*	0	less than 1 parasite

## Discussion

The ddPCR assay described in the present research demonstrated high sensitivity and specificity. Setting the annealing temperature to 50 °C does not represent a limit for the detection of *L. tarentolae*, as the non-specific population of droplets is clearly distinguishable from the positive droplets of interest. The limit of detection for *L. tarentolae* corresponds to 0.2 *Leishmania* cells/μl of dog blood, or, in other words, to one *Leishmania* cell included in the reaction mix, therefore, the assay can represent a powerful tool for estimating parasite prevalence in extensive epidemiological screenings.

Rain was observed in all samples. As our aim was to develop an assay for large epidemiological screenings, we kept a “conservative” threshold in terms of the amplitude of droplets that have to be considered positive. Excluding the rain by raising the threshold for positive droplets lowers the concentration detected by the assay. However, even by setting the threshold in a way in which all rain is excluded, the limit of detection of the assay does not change.

Our assay was characterized by minimal cross-reactivity of the probes with the non-target *Leishmania* species and with the controls represented by dog and sand fly DNA. The complete absence of cross-hybridization with the non-targets was observed in every experimental condition for the HEX probe targeting *L. infantum*. Limited hybridization of the FAM probe, targeted on *L. tarentolae*, occurred with non-targets instead. Indeed, up to four positive droplets were obtained analysing *L. infantum* samples and the controls of *Leishmania*-free dog blood and male sand flies. Therefore, in our assay, a result with fewer than five FAM-positive droplets and zero HEX-positive droplets should be classified as negative.

Of the 15 female *Leishmania*-positive sand fly samples tested with the ddPCR assay, 11 were consistent with the qPCR results obtained previously, using ITS-1 as the target [31]. Two *S. minuta* sand flies resulted positive only to *L. tarentolae* after qPCR, but also resulted positive to *L. infantum* after ddPCR, with a post-amplification target DNA concentration of less than one copy/μl. This result suggests a possible contact of these sand flies with *L. infantum* parasites, with the corresponding molecular signature undetected after the ITS-1-targeted qPCR. The ddPCR assay, instead, successfully revealed this signature, likely due to the use of kDNA minicircles as the target of the assay. These mitochondrial elements occur in thousands of copies per parasite [32], in contrast to the ITS-1 nuclear region, which is present in only 20 to 200 copies per cell [33]. This marked difference in copy number translates into a higher sensitivity of ddPCR, particularly when dealing with samples harbouring low parasite loads. The simultaneous detection of *L. tarentolae* and *L. infantum* in the tested sand flies is also in accordance with the finding of co-infected sand flies coming from the same area where the samples were collected [7]. One *S. minuta* and one *P. perniciosus* sand fly tested positive for *L. tarentolae* after ITS-1-targeted qPCR; however, after ddPCR application, the same samples were positive for *L. infantum* and negative for *L. tarentolae*. This incongruence may reflect mitonuclear discordance occurring in the *Leishmania* parasites infecting these sand flies. In mitonuclear discordance, nuclear and mitochondrial genomes have discordant evolutionary histories; this causes topological discordance between nuclear gene and mitochondrial gene-based phylogenies, ultimately resulting in incorrect species identification [34]. Mitonuclear discordance is the result of genetic exchange between different species, resulting in the formation of hybrids. The biological mechanism on the basis of mitonuclear discordance in *Leishmania* remains poorly understood, but there is experimental evidence that the genetic exchange between the parental species happens in the sand fly vector [34]. Therefore, we could interpret the discordance in the results we obtained as an example of mitonuclear discordance, occurring in two out of the 15 sand flies constituting the dataset tested in our study (frequency = 13.3%), with nuclear DNA (ITS-1) of *L. tarentolae* and mitochondrial DNA (kDNA) of *L. infantum*. A study in Peru described a similar frequency of New World *Leishmania* hybrids in samples obtained from CL human patients [35]. Our study provides the first evidence to date of mitonuclear discordance and possible hybridization between the representatives of two subgenera of the *Leishmania* genus, namely *L.* (*Leishmania*) and *L.* (*Sauroleishmania*). This also represents the first possible report of the phenomenon observed in wild-caught sand flies. The sympatric occurrence of *L. infantum* and *L. tarentolae* may enhance the possibilities of hybridization of these two *Leishmania* species [12]. This may have happened in the collection area of the sand flies tested in our study, in which the two *Leishmania* species coexist. Therefore, the use of both mitochondrial- and nuclear-targeted detection methods is recommended in areas where more than one species is present.

The method of ddPCR does not require the construction of a standard curve because it relies on the absolute quantification of the target molecules. Nevertheless, serial dilutions can be useful to experimentally determine the assay limit of detection. By comparing the ddPCR quantification of the serial dilution of *Leishmania* cells in dog blood, we can perform a broad estimation of the parasite cell number infecting the tested sand flies. The value of the concentration of *L. tarentolae* in three sand fly samples reached saturation (i.e. 1,000,000 copies of target/μl). This result is in accordance with the ITS-1 targeted qPCR cqs of these samples, which are amongst the lowest in the dataset. While preparing serial dilutions of *L. tarentola*e isolates employed in the ddPCR validation, the saturation was reached starting from 100,000 *L. tarentolae* cells (corresponding to 5000 parasite cells added in the reaction mix; not shown) spiked in dog blood. Therefore, the sand flies representing the above-mentioned samples could have harboured 5000 or more *Leishmania* cells. One sand fly sample showed a concentration higher than 10,000 *L. tarentolae* cells (corresponding to 500 parasite cells added in the reaction mix; not shown). The number of parasites estimated for the other samples ranged from less than 1 to 250 parasites. The estimated parasite count of less than one cell in some samples may result from mere contact between the sand fly and parasite DNA, rather than indicating an established infection. Indeed, these samples showed higher cqs when tested with the ITS-1 targeted qPCR. Overall, the estimation of *Leishmania* cells in sand flies, although being approximate, is consistent with the quantifications reported in the literature [36–38].

However, our ddPCR assay suffers from some limitations, some of them intrinsic to the method. The ddPCR is more expensive than the qPCR. For instance, in qPCR the cost is approximately $1.70 per sample, while in ddPCR the cost for a single sample is ∼$5.00. Moreover, ddPCR may be more prone to contamination than qPCR. Considering that ddPCR can detect minimal amounts of target DNA, the higher risk of contamination compared to qPCR is a disadvantage in the studies of *Leishmania* prevalence in sand flies. These insects can be collected and stored with methods by which they can come in close contact (i.e. sticky traps), and contamination may occur. Therefore, the assay may ultimately result in lower specificity due to a higher number of false-positive samples. To overcome this problem, as the limit of detection is as low, an experimental threshold should be set in order to identify possible cross-reactions, especially with other sand fly and *Leishmania* species. In fact, in this study, we were able to validate the assay only on *P. perniciosus* and *S. minuta* sand flies, and we did not include other *Leishmania* species apart from *L. tarentolae* and *L. infantum.*

## Conclusions

In this study, we present the first ddPCR protocol developed for the simultaneous detection of *L. tarentolae* and *L. infantum* parasites in both reservoir hosts and vectors. The assay exhibited high sensitivity, detecting low levels of DNA of both *L. tarentolae* and *L. infantum* parasites spiked into dog blood samples. This demonstrates the assay’s utility for reliable *Leishmania* detection in dog blood, underscoring its potential as a valuable tool for clinical diagnostics and early intervention in canine leishmaniasis. To our knowledge, this is the first ddPCR assay validated for use on sand flies. By enabling accurate detection of *Leishmania* in sand fly populations, this assay could significantly enhance monitoring efforts and inform targeted control strategies in endemic regions. This dual application makes the protocol highly valuable for comprehensive surveillance in both canine hosts and sand flies. To prevent misinterpretation of results, we highlight the importance of establishing an experimental threshold, particularly for *L. tarentolae*, to avoid false positives. Additionally, we recommend that screening for *Leishmania* parasites include both mitochondrial and nuclear targets to account for potential mitonuclear discordance.

## Data Availability

All the data generated in the paper are available in the paper itself.
